# Chemical Composition of Essential Oil from Flower of ‘Shanzhizi’ (*Gardenia jasminoides* Ellis) and Involvement of Serotonergic System in Its Anxiolytic Effect

**DOI:** 10.3390/molecules25204702

**Published:** 2020-10-14

**Authors:** Nan Zhang, Mu Luo, Lei He, Lei Yao

**Affiliations:** 1 Department of Landscape Architecture, School of Design, Shanghai Jiao Tong University, 800 Dong Chuan Road, Shanghai 200241, China; fxzwzhangnan@sjtu.edu.cn (N.Z.); reverie@sjtu.edu.cn (M.L.); 2 Department of Resources and Environment, School of Agriculture and Biology, Shanghai Jiao Tong University, 800 Dong Chuan Road, Shanghai 200241, China; 018150910007@sjtu.edu.cn

**Keywords:** *Gardenia jasminoides*, essential oil, inhalation, anxiolytic effect, serotonergic neurotransmission

## Abstract

*Gardenia jasminoides* Ellis is a famous fragrant flower in China. Previous pharmacological research mainly focuses on its fruit. In this study, the essential oil of the flower of ‘Shanzhizi’, which was a major variety for traditional Chinese medicine use, was extracted by hydro distillation and analyzed by GC-MS. Mouse anxiety models included open field, elevated plus maze (EPM), and light and dark box (LDB), which were used to evaluate its anxiolytic effect via inhalation. The involvement of monoamine system was studied by pretreatment with neurotransmitter receptor antagonists WAY100635, flumazenil and sulpiride. The monoamine neurotransmitters contents in the prefrontal cortex (PFC) and hippocampus after aroma inhalation were also analyzed. The results showed that inhalation of *G. jasminoides* essential oil could significantly elevated the time and entries into open arms in EPM tests and the time explored in the light chamber in LDB tests with no sedative effect. WAY100635 and sulpiride, but not flumazenil, blocked its anxiolytic effect. Inhalation of *G. jasminoides* essential oil significantly down-regulated the 5-HIAA/5-HT in the PFC and reduced the 5-HIAA content in hippocampus compared to the control treatment. In conclusion, inhalation of gardenia essential oil showed an anxiolytic effect in mice. Monoamine, especially the serotonergic system, was involved in its anxiolytic effect.

## 1. Introduction

*Gardenia jasminoides* Ellis (*Rubiaceae*) is native to subtropical regions of East Asia. It is a very famous fragrant flower in China. It is widely distributed and cultivated in most of the provinces and regions south of the Yangtze River. Among them, ‘Shanzhizi’ (*G. jasminoides*) and ‘Shuizhizi’ (*G. jasminoides* forma *longicarpa* Z.W. Xie et Okada.) are the most common cultivars. Different from the common garden ornamental varieties, ‘Shanzhizi’ is single-flowered. It is a major variety in the Chinese medicine planting industry. Previous pharmacological research mainly focused on its fruit for it had been used in traditional Chinese medicine for a long history. The extract of fruit showed potential neuroprotective effects [1], memory-enhancing capacity [2], and anti-inflammatory effects [3], in several pharmacological research projects. Moreover, the flowers and roots of *G. jasminoides* are also used as medicines in folk medicine. In southern China, the flower of *G. jasminoides* is often used as a vegetable for soups, stir-fries, and cold salads.

At present, only a few research projects have focused on the potential effects of the volatile components of the flower. Several papers have reported on the antibacterial activities of *G. jasminoides* flower essential oil [4,5]. In this study, we focus on the volatile components of the essential oil of the flower of ‘Shanzhizi’. The essential oil of the ‘Shanzhizi’ flower was extracted by hydro distillation and the chemical components were analyzed by GC-MS. Animal anxiety behavior models such as elevated plus maze, open field, and light and dark box were used to evaluate the anxiolytic effect of the aroma. The sedative effect of the aroma was also evaluated. The involvement of monoamine system was studied by pretreatment with three neurotransmitter receptor antagonists and the monoamine neurotransmitters contents in different brain regions after aroma inhalation were also analyzed.

## 2. Material and Method

### 2.1. Plant Material and Essential Oil Preparation

The flowers of ‘Shanzhizi’ (*G. jasminoides*) were collected in May 2019 in Zhejiang province of China. The *G. jasminoides* essential oil (GEO) was extracted from the fresh flower of ‘Shanzhizi’ by hydro distillation. One Kg of ‘Shanzhizi’ flowers and 3 L of water were added to a 5 L distillation device, followed by an ultrasonic treatment for 20 min and a constant temperature distillation for 4 h. The upper essential oil in the oil-water separator was collected. From 1 kg of fresh flowers, 0.07 g of essential oil could be extracted on average.

### 2.2. Animals

Male ICR (Institute of Cancer Research) mice were purchased from the Shanghai SLAC Laboratory Animal Co., Ltd. (Shanghai, China). The using of animals and experimental procedures were performed following the rules of the Association for Assessment and Accreditation of Laboratory Animal Care International and approved by the Institutional Animal Care (Frederick, MD, USA) and Use Committee of Shanghai Jiao Tong University (Shangai, China). Mice were housed in cages under a 12/12 h light/dark cycle and controlled temperature (21 ± 1 °C) with free food and water. At the beginning of experiment, the weights of the mice were 25–30 g. (The study was approved by the Institutional Animal Care and Use Committee (IACUC) of Shanghai Jiao Tong University, the ID was A2018051.The date of approval is 2018-7-24.)

### 2.3. Chemical and Treatment

Diazepam (DZP) injection (Shanghai Xudong Haipu Pharmaceutical Co., LTD., Shangai, China) diluted with saline was intragastric administrated to mice 30 min before the behavioral test as a positive control drug. WAY100635 (WAY, 5-HT1A antagonist, Sigma-Aldrich, St. Louis, MI, USA) and flumazenil (FLU, benzodiazepine binding antagonist, Aladdin, China) were dissolved in saline. Sulpiride (SUL, dopamine D2/D3 receptor antagonist, Aladdin, China) was dissolved in tween 80-saline solution (1%). DZP (1 mg/kg), WAY100635 (0.3 mg/kg), FLU (5 mg/kg), and SUL (40 mg/kg) were prepared freshly and were injected intraperitoneally (i.p.) in mice at 0.1 mL/10 g of body weight 30 min before the behavioral test. Sodium pentobarbital was diluted with saline and intraperitoneal injection to the mice at a dose of 50 mg/kg.

The GEO was diluted by camellia seed oil (provided by the Aromatic Plant R & D center of Shanghai Jiao Tong University, Shanghai, China) to 10% (*v*/*v*). Three concentrations (0.2 mg/L, 1.0 mg/L, 2 mg/L) of odor were established as the inhalation environment. Pure camellia seed oil was used as the control treatment.

### 2.4. Inhalation Apparatus

The inhalation apparatus was composed of a lower chamber (18 × 24 × 25 cm) and an upper chamber (18 × 24 × 25 cm). The essential oil was diffused by an ultrasonic diffuser, which were placed in the lower chamber. There was an iron plate between the two chambers with a circular hole in the middle. A short section of plastic tube was used to connect the diffuser and the upper chamber, which allowed the fragrance that volatilized from the ultrasonic diffuser pass into the upper chamber. Experimental animals were placed in the upper chamber and could explore the space freely.

### 2.5. Identification of the Constituents of Essential Oil

The essential oil was diluted 10 times with a solution of ethanol and n-hexane (1:1, *v*/*v*) and then directly injected the sample for detection. Gas chromatography/Mass spectrometer (GC/MS, Agilent 7890B-5977A) was used to analyze the constituents of GEO. The GC was filled with a methylpolysiloxane nonpolar column (HP-5: 30 m × 0.32 mm × 0.25 μm). The GC conditions were as follows: Carrier gas, helium (1 mL/min); split rate, 10:1; column temperature, 50 °C for 10 min, 50 °C to 220 °C at 4 °C/min, then 220 °C for 10 min, 220 °C to 280 °C at 20 °C/min, then 280 °C for 3 min. The MS conditions were as follows: Inlet line temperature, 280 °C; source temperature, 230 °C; mass spectra electron impact, 70 eV. Individual components were identified from the mass spectral library (NIST14).

### 2.6. Behavioral Test

#### 2.6.1. Open Field Test

In the open field (OF) test, mice were individually placed in the center of a black square area (45 × 45 cm) with apparent walls (40 cm) around. Mice were allowed to explore the area for 5 min. The total distance was recorded and analyzed by a camera above the test area.

#### 2.6.2. Elevated Plus Maze Test

The elevated plus maze (EPM) consisted of two open arms (30 × 6 cm, length × width) and two closed arms (30 × 6 × 15 cm, length × width × height) connected by an open central platform (6 × 6 cm, length × width). The maze was elevated 55 cm above the floor and placed inside a sound-attenuated room. Mice were individually placed on the platform with head toward the open arms, and allowed to freely explore for 5 min. The total amount of time the mice spent in open arms or closed arms were recorded during the 5 min test period. The percentage of the time spent in open arms was calculated with the following formula: the time spent in open arms/the total time spent in open and closed arms × 100. Additionally, the numbers of entries into these arms were also recorded. The percentage of the open arm entries was calculated with the following formula: the number of entries into the open arms/the total number of entries into the open and closed arms × 100.

#### 2.6.3. Light and Dark Box Test

The apparatus consisted of two chambers with lid divided by a connection door (5 × 5 cm). The light chamber (27 × 18 × 18 cm) was entirely white and illuminated with 400-lux light shining. The dark chamber (18 × 18 × 18 cm) was entirely black. Both chambers were equipped with a camera above. Each mouse was individually placed in the light chamber facing the door, and allowed to freely explore the apparatus for 5 min. The time spent in the light chamber were recorded.

#### 2.6.4. Pentobarbital Sodium Sleep Test

Following sodium pentobarbital injection, the mice were individually placed in a breeding cage for observation, and the behavior of the mice was recorded through a camera. The time from the time of administration to the disappearance of the righting reflex was recorded as the sleep latency, while the time from the disappearance of righting reflex to recovery was recorded as the duration of sleep time.

### 2.7. Quantification of Monoamines and Their Metabolites

Immediately after the behavioral test, mice were decapitated. The prefrontal cortex (PFC) and hippocampus were dissected and stored at −80 °C. The sample pretreatments and detection methods were the same as the previous report [6]. Samples were ultrasound homogenized in a solution containing 0.6 mol/L perchloric acid, 0.5 mmol/L EDTA-Na_2_ and 0.1 g/L L-cysteine. The homogenates were centrifuged at 10,000 rpm for 20 min at 4 °C. The supernatants were then centrifuged with sinking agent containing 1.2 M dipotassium phosphate and 2 mM EDTA-Na_2_ at 12,000 rpm for 15 min at 4 °C and stored at 80 °C. Norepinephrine (NE), dopamine (DA), 5-hydroxytryptamine (5-HT) and 5-hydroxyindoleacetic acid (5-HIAA) were measured by high performance liquid chromatography with a fluorescence detector (HPLC/FLD, e2695/2475, Waters, New York, NY, USA). Syncronis C18 column (250 × 4.6 mm, 5 µm, Thermo, Waltham, MA, USA) was used. Methanol-water (13:87, *v*/*v*) was used as mobile phase (pH 3.8) and delivered at 0.8 mL/min. Sodium acetate (50 mM) and citric acid (50 mM) were used as pH buffer. Sodium 1-heptanesulfonate (0.5 mM) and EDTA-Na_2_ (5 mM) were used as ion pairing agent. Excitation and emission of the fluorescence detector were 280 and 330 nm, respectively. Acquisition and integration of chromatograms were performed using the Empower software. The concentrations of neurotransmitters were calculated by the calibration curve of external standards.

### 2.8. Experimental Procedures

#### 2.8.1. Effects of Odor Exposure on Anxiety Models and Pentobarbital Sodium Sleep Model

Mice were placed in the upper chamber individually for a 10 min-inhalation after 30 min habitation in the test room. Their behaviors in behavioral tests were examined immediately after odor exposure. Some of the mice performed OF and EPM tests, some of the mice performed the LDB test, and the other mice performed the pentobarbital sodium sleep experiment. The OF test was performed first followed by the EPM test. DZP was used as the positive drug. Mice were sacrificed by decapitation immediately after the EPM test. The prefrontal cortex and hippocampus were dissected for a further study. All experimental procedures occurred between 10:00 a.m. and 5:00 p.m.

#### 2.8.2. Study of the Involvement of Monoamine System

Antagonists (WAY, SUL and FLU) or saline were individually administered (i.p.) 20 min prior to a 10 min GEO odor exposure or control treatment. Behavioral tests (OF and EPM) were held immediately after the odor exposure. All experimental procedures occurred between 10:00 a.m. and 5:00 p.m.

### 2.9. Statistical Analyses

Animals’ behaviors were analyzed using the software XR-Xmaze (Xinruan information technology Co., Ltd., Nanjing, China). One-way ANOVA followed by a post hoc Duncan test was used to analysis the mice behaviors. Unpaired two-tailed Student’s *t*-test was used to analysis the neurotransmitters data.

## 3. Results

### 3.1. The Identification of Major Constituents of G. jasminoides Flower Essential Oil

**Figure 1:**
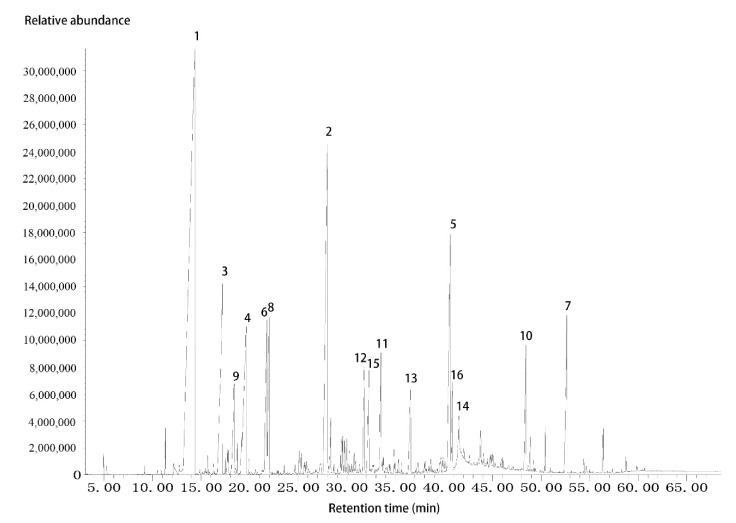
Total ion gas chromatogram of *Gardenia jasminoides* essential oil. Peaks: (1) Linalool; (2) α-farnesene; (3) α-terpineol; (4) geraniol; (5) cembrene A; (6) cis-3-hexenyl tiglate; (7) pentacosane; (8) hexyl tiglate; (9) nerol; (10) tricosane; (11) geranyl angelate; (12) tau.-cadinol; (13) 8-hydroxylinalool; (14) n-hexadecanoic acid; (15) α-terpinyl acetate; (16) verticiol.

The constituents of GEO were analyzed by GC/MS. There were 16 ingredients with relative content greater than 1% in the essential oil, which account for 83.95% of the total (Figure 1). These compounds were listed in the Table 1. The MS data of these compounds could be found in Supplementary Figure S1. Terpenes, alcohols and esters were the main components of GEO. Linalool (34.7%), α-farnesene (10.2%), α-terpineol (6.3%), geraniol (5.8%) and cembrene A (5.8%) were the main components of the essential oil. cis-3-Hexenyl tiglate (3.1%), hexyl tiglate (2.4%) and geranyl angelate (1.9%) were the three main esters in the essential oil.

### 3.2. Effects of GEO Exposure on Anxiety Animal Models

**Figure 2:**
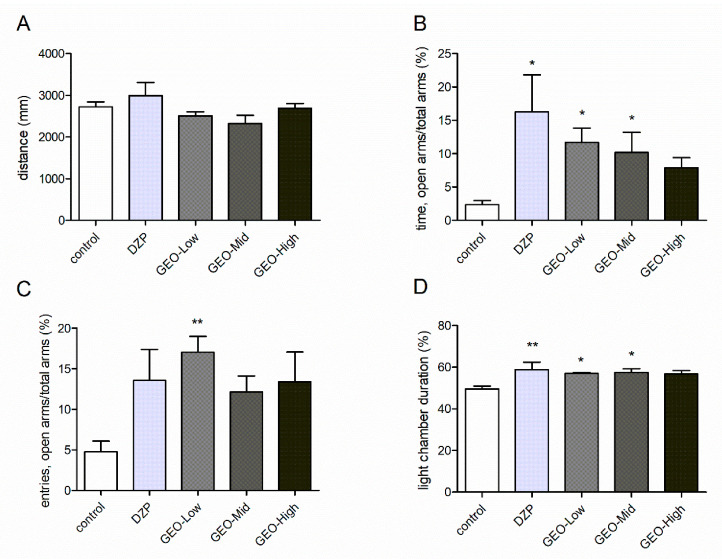
Anxiolytic effects of GEO odor under three concentrations (GEO-Low: 0.2 mg/L, GEO-Mid: 1.0 mg/L, GEO-High: 2.0 mg/L) exposures on the mice in the open field (OF) test (**A**), elevated plus maze (EPM) test (**B**,**C**) and light and dark box (LDB) test (**D**). (**A**) The total distance of the mice explored in the OF test. (**B**) The percentage of time of the mice spent in open arms in EPM tests. (**C**) The percentage of entries of the mice into the open arms in EPM tests. (**D**) The percentage of time spent in the light chamber in LDB tests. Values represent the mean ± SE (*n* = 10~11), * *p* < 0.05 vs. control group, ** *p* < 0.01 vs. control group. A one-way ANOVA followed by a post hoc Duncan test was used.

In the OF test, treatments did not affect the behavior of the mice (*F* (4, 46) = 1.85, *p* > 0.05). Compared to the control treatment, none of the treatment changed the total distance of the mice explored in the OF test (Figure 2A).

In the EPM test, odor exposure significantly affected the behaviors of the mice (*F* (4, 46) = 2.83, *p* < 0.05). The positive drug DZP significantly increased the percentage of time of mice spent in the open arms (*p* < 0.05). Similarly, the low and mid doses of GEO treatments significantly elevated the time of mice spent on the open arms from 2.4% to 11.7% and 10.2% (*p* < 0.05), respectively, compared to the control group (Figure 2B). Moreover, the low dose of GEO significantly elevated the entries spent into the open arms from 4.8% to 17.0% (*p* < 0.05, Figure 2C).

In the LDB test, odor exposure showed an anxiolytic effect (*F* (4, 46) = 3.22, *p* < 0.05). The low and mid dose treatments of GEO elevated the time duration in the light chamber from 49.6% to 57.0% and 57.5% respectively compared to the control group (Figure 2D). The positive drug DZP also significantly increased the percentage of time of mice spent in light chamber from 49.6% to 58.8% (*p* < 0.05).

### 3.3. Effects of GEO Exposure in the Pentobarbital Sodium Sleep Test

**Figure 3:**
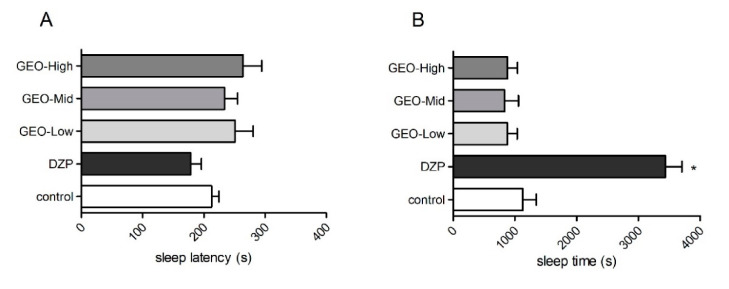
The effects of GEO odor exposures on the mice in pentobarbital sodium sleep test. Sleep latency (**A**) and sleep time (**B**) after GEO exposure with three concentrations (GEO-Low: 0.2 mg/L, GEO-Mid: 1.0 mg/L, GEO-High: 2.0 mg/L) were recorded. Values represent the mean ± SE (*n* = 8), * *p* < 0.05 vs. control group. One-way ANOVA followed by a post hoc Duncan test was used.

The pentobarbital sodium sleep test was used to test the sedative effect of the GEO exposure (Figure 3). The results showed that DZP significantly increased the sleep time of the mice compared to the control treatment (*F* (4, 35) = 29.11, *p* < 0.05). None of the three doses of GEO exposure affected the sleep time or sleep latency of the mice compared to the control treatment (*p* > 0.05).

### 3.4. Effects of Pretreatment of WAY100635, Sulpiride and Flumazenil on the Anxiolytic Effect of GEO

**Figure 4:**
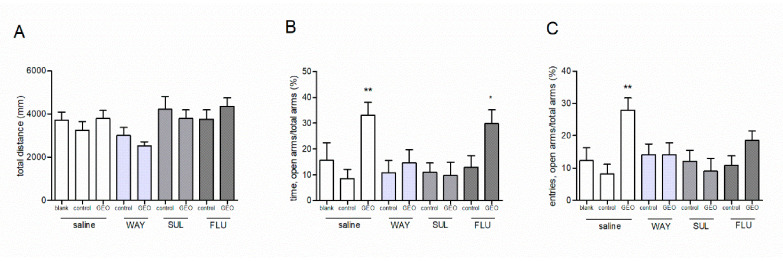
Effects of pretreatment of WAY100635, sulpiride and flumazenil on the anxiolytic effect of GEO (0.2 mg/L) in OF and EPM tests. (**A**) The total distance of the mice explored in the OF test. (**B**) The time the mice spent in open arms in EPM tests. (**C**) The entries of the mice into the open arms in EPM tests. WAY: WAY100635 (0.3 mg/kg), SUL: Sulpiride (40 mg/kg), FLU: Flumazenil (5 mg/kg). Values represent the mean ± SE (*n* = 8), * *p* < 0.05 vs. saline + control group, ** *p* < 0.01 vs. saline + control group. One-way ANOVA followed by a post hoc Duncan test was used.

A low dose (0.2 mg/L) of GEO exposure was used in this study. Pretreatment of three neurotransmitter antagonists did not significantly affected the total explored distance of the mice in the OF test (*F* (8, 63) = 2.07, *p* > 0.05). None of the antagonists significantly change the behaviors of mice after GEO treatment compared to the control treatments (*p* > 0.05, Figure 4A).

In the EPM test, some of the neurotransmitter antagonists showed a significant effect on the anxiolytic effect of GEO (the percentage of time in open arms: *F* (8, 63) = 3.28, *p* < 0.05; the percentage of entries into open arms: *F* (8, 63) = 2.95, *p* < 0.05). When pre-treated with saline, GEO significantly elevated the percentage of time and entries in the open arms compared to the control treatment (*p* < 0.05, Figure 4B,C). There was no significant difference in behaviors between the control group and the blank group (no odor exposure treatment). This result indicated that camellia seed oil exposure showed no effect on the mice anxiety behaviors in the EPM test. GEO pre-treated with WAY and SUL treatments showed no effect on the anxiety behaviors compared to the control + saline treatment (*p* > 0.05, Figure 4B,C). GEO pre-treated with FLU still elevated the percentage of time spent in the open arms compared to the control + saline treatment (*p* < 0.05). These results above indicated that WAY and SUL, but not FLU, blocked the anxiolytic effect of GEO.

### 3.5. Effects of GEO on the Monoamine Neurotransmitters in Two Brain Regions

**Figure 5:**
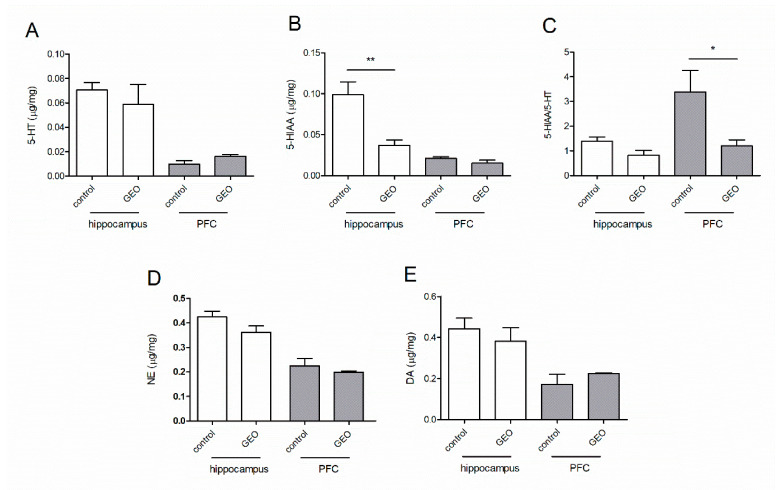
Changes in the contents of monoamine neurotransmitters in the prefrontal cortex and hippocampus of the mice after GEO exposure (0.2 mg/L). (**A**–**C**) Contents of 5-HT (**A**), 5-HIAA (**B**) and the ratio of 5-HIAA/5-HT (**C**) in the hippocampus and PFC. (**D**,**E**) Contents of NE (**D**) and DA (**E**) in the hippocampus and PFC. Values represent the mean ± SE (*n* = 5), * *p* < 0.05, ** *p* < 0.01; unpaired two-tailed Student’s *t*-test was used.

GEO exposure (0.2 mg/L) affected the 5-HIAA content in the hippocampus of the mice (Figure 5B). The 5-HIAA content in GEO exposure group (0.099 μg/mg) was significantly higher (*p* < 0.05) than in the control group (0.036 μg/mg). GEO also elevated the 5-HT content and decreased the 5-HIAA content in the PFC of the mice compared to the control treatment though it did not show a statistical difference (Figure 5A,B). The 5-HIAA/5-HT ratio of the control group was higher than in the GEO group in the hippocampus and PFC, and it showed a statistical difference between the control and GEO group in PFC (*p* < 0.05). These results indicated that GEO changed the metabolic rate of 5-HT in the PFC (Figure 5C). There were no significant differences on the NE and DA content in the GEO and control group (Figure 5D,E).

## 4. Discussion

Unlike previous studies that focused on the pharmacological effects of *G. jasminoides* fruit extracts, this study focused on the neuropharmacological functions of the aroma of flowers. *G. jasminoides* is a traditional Chinese fragrant flower, which is deeply loved by the public and widely used in gardening. Its organic solvent extract and essential oil have appeared in aromatherapy market in recent years. We used ‘Shanzhizi’ (*G. jasminoides*) as the research object because it has a large planting area in China as a raw material for traditional Chinese medicine. The chemical components of the essential oil of fresh ‘Shanzhizi’ flowers were mainly terpene alcohols and terpenes, in which linalool and α-farnesene were the main components. According to our previous review of anxiolytic ingredients of essential oil [7], essential oil with such chemical components might have certain anxiolytic activity.

In the following EPM and LDB tests, *G. jasminoides* essential oil showed expected anxiolytic activity. Three doses of GEO were tested. The low-dose odor showed significant anxiolytic activity in both EPM and LDB tests. However, a high-dose odor did not show significant anxiolytic effects in either of the two tests. Such difference induced by odor concentration was found in many previous studies [8,9]. The information processing and coding mode of odor information might be one of the reasons for this phenomenon [10]. In the pentobarbital sodium sleep test, GEO exposure did not prolong the sleep time of the mice, which indicated that GEO has no sedative effect. In a previous study, many essential oils, such as lavender oil [11,12] and lemon oil [13,14], and chemicals, such as linalool [9,15] and limonene [16,17], showed both anxiolytic and sedative effects through inhalation administration. Inhalation of linalool under an anxiolytic dose could also decrease the total distance the mice explored in the OF test [9]. In the present study, inhalation of GEO containing linalool did not affect the behavior in the OF test and exerted no sedative effect in the pentobarbital sodium sleep test. Antagonism between chemicals might be one of the reasons for this phenomenon.

To investigate involvements of the monoamine neurotransmitter system in the anxiolytic effect of GEO, three neurotransmitter receptor antagonists were pretreated before the odor exposure and the monoamine neurotransmitters contents in different brain regions after aroma inhalation were analyzed. WAY100635, a highly selective 5-HT1A antagonist, was reported to exhibit a blocking effect on the anxiolytic effect induced by some essential oils, such as lavender oil, which was thought to have an anxiolytic effect through serotonergic but not GABA_A_/benzodiazepine neurotransmission [18]. Sulpiride, a dopamine D2/D3 receptor antagonist, was reported to block the antidepressant-like effect of marjoram oil [19]. Flumazenil, a competitive antagonist of benzodiazepine binding, has been demonstrated to exhibit antagonism on (+)-limonene epoxide [20], linalool [21] and phytol [22]. In this study, WAY100635 and sulpiride, but not flumazenil, blocked the anxiolytic effect of GEO. This result indicates that serotonergic, dopaminergic but not GABA_A_/benzodiazepine neurotransmission might be involved in the anxiolytic effect of GEO. The quantitative results of neurotransmitters showed that inhalation of GEO significantly down-regulated the 5-HIAA/5-HT ratio in the PFC and reduced the 5-HIAA content in the hippocampus compared to the control treatment. These results further confirmed that the serotonergic neurotransmission was involved in the effect of GEO.

## Figures and Tables

**Table 1:** Main constituents of *G. jasminoides* essential oil (GEO).

Peak No.	Retention Time (Min)	Compound	Peak Area (%)
1	14.35	Linalool	34.7
2	28.00	α-Farnesene	10.2
3	17.19	α-Terpineol	6.3
4	19.66	Geraniol	5.8
5	40.67	Cembrene A	5.8
6	21.78	cis-3-Hexenyl tiglate	3.1
7	52.66	Pentacosane	3.0
8	21.99	Hexyl tiglate	2.4
9	18.41	Nerol	2.0
10	48.44	Tricosane	1.9
11	33.51	Geranyl angelate	1.9
12	31.79	tau.-Cadinol	1.8
13	36.57	8-Hydroxylinalool	1.7
14	41.55	n-Hexadecanoic acid	1.3
15	32.30	α-Terpinyl acetate	1.2
16	40.86	Verticiol	1.0
Sum			84.0

## Supplementary Materials

The following are available online, Figure S1: The MS data of 16 main components in the essential oil of *Gardenia jasminoides.* A, linalool; B, α-farnesene; C, α-terpineol; D, geraniol; E, cembrene A; F, cis-3-hexenyl tiglate; G, pentacosane; H, hexyl tiglate; I, nerol; J, tricosane; K, geranyl angelate; L, tau.-cadinol; M, 8-hydroxylinalool; N, *n*-hexadecanoic acid; O, α-terpinyl acetate; P, verticiol.
