# Supplementary material for: Chemical Composition of Essential Oil from Flower of ‘Shanzhizi’ (Gardenia jasminoides Ellis) and Involvement of Serotonergic System in Its Anxiolytic Effect
Source: Molecules. 2020 Oct 14;25(20):4702. doi: 10.3390/molecules25204702 (PMC7587363; doi:10.3390/molecules25204702)
Supplement: Supplementary file 1 [file molecules-25-04702-s001.pdf]

molecules

Chemical Composition of Essential Oil from Flower of 'Shanzhizi' (*Gardenia jasminoides* Ellis) and Involvement of Serotonergic System in Its Anxiolytic Effect

Nan Zhang [1], Mu Luo [1], Lei He [2] and Lei Yao [1,*]

[1] Department of Landscape Architecture, School of Design, Shanghai Jiao Tong University, 800 Dong Chuan Road, Shanghai 200241, China; fxzwzhangnan@sjtu.edu.cn (N.Z.); reverie@sjtu.edu.cn (M.L.)

[2] Department of Resources and Environment, School of Agriculture and Biology, Shanghai Jiao Tong University, 800 Dong Chuan Road, Shanghai 200241, China; 018150910007@sjtu.edu.cn

* Correspondence: yaolei@sjtu.edu.cn; Tel./Fax: +86-21-34206606

Supplementary Materials

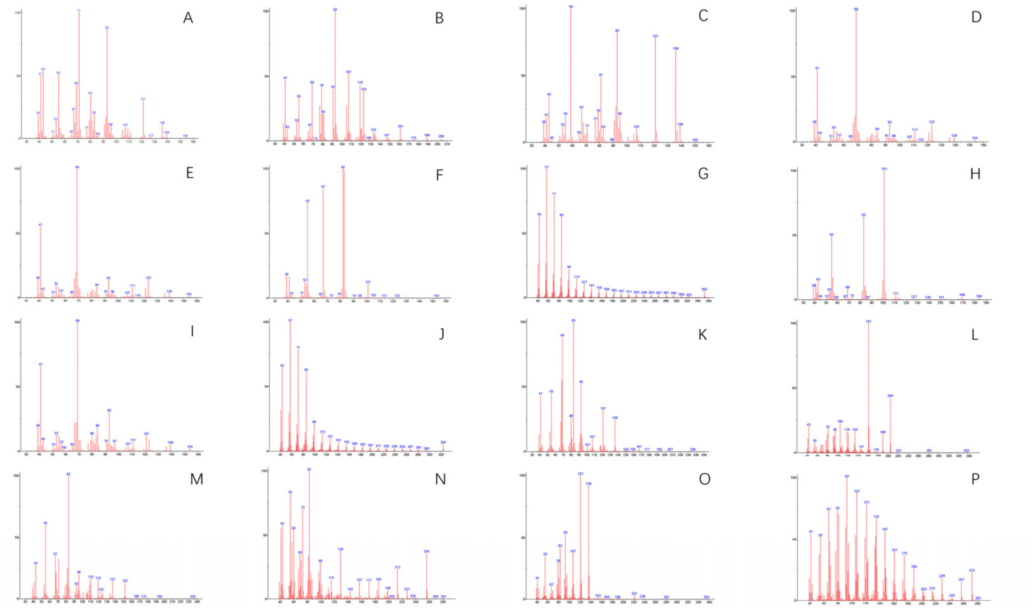

Figure S1. The MS data of 16 main components in the essential oil of *Gardenia jasminoides.* A, linalool; B, α-farnesene; C, α-terpineol; D, geraniol; E, cembrene A; F, cis-3-hexenyl tiglate; G, pentacosane; H, hexyl tiglate; I, nerol; J, tricosane; K, geranyl angelate; L, tau.-cadinol; M, 8-hydroxylinalool; N, n-hexadecanoic acid; O, α-terpinyl acetate; P, verticiol.
